# Automatic Perception of Typical Abnormal Situations in Cage-Reared Ducks Using Computer Vision

**DOI:** 10.3390/ani14152192

**Published:** 2024-07-27

**Authors:** Shida Zhao, Zongchun Bai, Lianfei Huo, Guofeng Han, Enze Duan, Dongjun Gong, Liaoyuan Gao

**Affiliations:** 1 Institute of Agricultural Facilities and Equipment, Jiangsu Academy of Agricultural Sciences, Nanjing 210014, China; 2 Key Laboratory of Protected Agriculture Engineering in the Middle and Lower Reaches of Yangtze River, Ministry of Agriculture and Rural Affairs, Nanjing 210014, China; 3 College of Engineering, Huazhong Agricultural University, Wuhan 430070, China; 4 State Key Laboratory of Intelligent Agricultural Power Equipment, Luoyang 471039, China

**Keywords:** cage-reared, meat duck, attention mechanism, abnormal detection, pose estimation

## Abstract

**Simple Summary:**

Efficient breeding of meat ducks using three-dimensional and multi-layer cages is a novel approach being actively explored in China. In this process, timely and accurate detection of abnormal situations among ducks is crucial for optimizing and refining the cage-rearing system, and ensuring animal health and welfare. This study focused on the overturned and dead status of cage-reared ducks using YOLOv8 as the basic network. By introducing GAM and Wise-IoU loss functions, we proposed an abnormal-situation recognition method for cage-reared ducks based on YOLOv8-ACRD. Building on this, we refined the identification of key body parts of cage-reared ducks, focusing on six key points: head, beak, chest, tail, left foot, and right foot. This resulted in the development of an abnormal posture estimation model for cage-reared ducks, based on HRNet-48. Furthermore, through multiple tests and comparative verification experiments, it was confirmed that the proposed method exhibited high detection accuracy, generalization ability, and robust comprehensive performance. The method proposed in this study for perceiving abnormal situations in cage-reared ducks not only provides foundational information for the progress and improvement of the meat duck cage-reared system but also offers technological references for the intelligent breeding of other cage-reared poultry.

**Abstract:**

Overturning and death are common abnormalities in cage-reared ducks. To achieve timely and accurate detection, this study focused on 10-day-old cage-reared ducks, which are prone to these conditions, and established prior data on such situations. Using the original YOLOv8 as the base network, multiple GAM attention mechanisms were embedded into the feature fusion part (neck) to enhance the network’s focus on the abnormal regions in images of cage-reared ducks. Additionally, the Wise-IoU loss function replaced the CIoU loss function by employing a dynamic non-monotonic focusing mechanism to balance the data samples and mitigate excessive penalties from geometric parameters in the model. The image brightness was adjusted by factors of 0.85 and 1.25, and mainstream object-detection algorithms were adopted to test and compare the generalization and performance of the proposed method. Based on six key points around the head, beak, chest, tail, left foot, and right foot of cage-reared ducks, the body structure of the abnormal ducks was refined. Accurate estimation of the overturning and dead postures was achieved using the HRNet-48. The results demonstrated that the proposed method accurately recognized these states, achieving a mean Average Precision (mAP) value of 0.924, which was 1.65% higher than that of the original YOLOv8. The method effectively addressed the recognition interference caused by lighting differences, and exhibited an excellent generalization ability and comprehensive detection performance. Furthermore, the proposed abnormal cage-reared duck pose-estimation model achieved an Object Key point Similarity (OKS) value of 0.921, with a single-frame processing time of 0.528 s, accurately detecting multiple key points of the abnormal cage-reared duck bodies and generating correct posture expressions.

## 1. Introduction

China is the largest global producer of duck meat. In recent years, the demand for duck meat and advancements in efficient breeding techniques have increased the annual number of slaughtered ducks. By 2023, China had 4.218 billion ducks, a 5.40% increase from 2022, with a total output value of 126.369 billion yuan, reaching a 5.04% increase from the previous year [1]. Traditionally, the duck meat industry in China has relied on extensive breeding methods such as free-range and open-field grazing, which are inefficient and cause severe environmental pollution. In response to governmental demands for sustainable livestock practices, intensive, efficient, and environment-friendly cage rearing has become the primary modern poultry breeding model [2]. During cage rearing, increased stocking density and reduced activity space, combined with the inherently sensitive and stress-prone nature of ducks, can often lead to abnormal behaviors, resulting in significant economic losses [3]. These behaviors include overturning and mortality. Overturning, often stress-induced, tends to occur around 10 days of age when the ducks’ skeletal development is insufficient to support their bodies, leaving them unable to right themselves without assistance. Without manual intervention, their physiological health is at risk. Moreover, deceased ducks, as carriers of infectious diseases, can cause large-scale breeding accidents if not promptly addressed. Therefore, efficient, accurate, and timely detection of abnormal situations in cage-reared ducks is crucial for ensuring healthy breeding practices and improving the cage-rearing mode.

Traditional screening for abnormal poultry primarily relies on manual inspection, which is time-consuming, labor-intensive, and highly subjective [4]. Additionally, this method can facilitate the cross-species transmission of pathogens. Although attaching acceleration sensors [5,6,7,8] and RFID [9,10,11] tags to poultry can automatically obtain motion information, this approach has drawbacks including high cost, cumbersome operation, and rejection by animals. Recently, with the continuous advancement of AI technology, many scholars have explored deep learning-based detection methods for poultry, utilizing various physiological features, such as appearance, behavior, sound, body temperature, and feces to address these issues.

Cui et al. [12] proposed a method for recognizing the health status of broiler chickens based on an improved YOLOv5 model, achieving an average accuracy of 97.80%. Zhuang et al. [13] leveraged differences in feather coverage and posture between healthy and diseased chickens, and optimized the Single Shot Multibox Detector (SSD) to enhance the accuracy and achieve precise classification of diseased and healthy chickens within a flock. Yang et al. [14] developed a method for calculating feather coverage on the backs of laying hens using thermal infrared images and the Otsu algorithm, elucidating the correlation between feather coverage and body temperature. Aydin [15] used depth cameras to collect 3D data on broilers, number of lying events (NOL), and latency to lie down (LTL) of lying behaviors. By comparing these data with previous records, a method was proposed for detecting limping in broiler chickens. Xiao et al. [16] achieved the precise semantic segmentation of caged chickens’ heads and body parts using binocular vision, conducting 3D reconstruction for the feeding movement analysis in three dimensions. Chai et al. [17] effectively monitored the floor egg-laying behavior in free-range laying hens using deep learning techniques. Cuan et al. [18] proposed an early detection method for Newcastle disease (ND) in poultry by analyzing the poultry’s sounds with deep learning networks, achieving a 98.50% accuracy rate. Shen et al. [19] adopted convolutional neural networks with infrared thermal imaging to extract the highest temperatures of broiler chickens’ heads and legs, creating an automatic temperature-detection model for white-feathered broilers. This model, incorporating environmental temperatures, relative humidity, and light intensity had an average relative error of only 0.29%. Degu et al. [20] developed a poultry disease discrimination method based on the images of diseased chicken feces, using the YOLOv3 and ResNet-50 target detection algorithms with smartphones, achieving a 98.70% classification accuracy for identifying health, coccidiosis, salmonella, and Newcastle disease. Gu et al. [21] proposed a method for real-time behavior recognition of cage-reared laying ducks by enhancing YOLOv4, offering a valuable technical reference for anomaly detection in cage-reared ducks.

Although these achievements have facilitated multi-target monitoring and disease diagnosis in broiler and layer chickens, there is a lack of research on methods for identifying abnormal situations in cage-reared ducks, necessitating innovative approaches. Given the strong learning and reasoning capabilities of computer vision technology in healthy poultry breeding, exploring its use in detecting and addressing abnormal situations in cage-reared ducks is both feasible and promising.

In current cage-rearing practices for meat ducks, a single cage typically holds 10–20 ducks (depending on cage size), each exhibiting varied physiological states and behavioral patterns. To ensure experimental consistency, this study specifically focused on ducks that were overturned or deceased, while disregarding others. In summary, this study addressed the demand for detecting abnormal conditions in cage-reared ducks, with a focus on 10-day-old ducks prone to such issues. This study consisted of four parts based on computer vision technology: (1) proposing an object detection model for over-turned and dead ducks in cages using an improved YOLOv8, (2) evaluating the impact of embedding global attention mechanism (GAM) into the original YOLOv8 network structure and replacing the CIoU loss function with Wise-IoU on the detection accuracy, (3) testing the generalization ability of the optimized model by adjusting image brightness levels (‘bright’ and ‘dark’) and comparing its performance with other mainstream methods, (4) introducing six key points, including head, beak, chest, tail, and left and right feet to refine the detection of abnormal situations in cage-reared ducks, and (5) proposing a method for abnormal cage-reared duck pose-estimation based on HRNet-48, and conducting performance comparison tests. The proposed method not only provides fundamental data for advancing cage-reared duck technology but also offers technical references for the intelligent breeding of other poultry species.

## 2. Materials and Methods

### 2.1. Image Acquisition and Experimental Material

In this study, 10-day-old Cherry Valley meat ducks raised at the Jiangsu Academy of Agricultural Sciences Animal Experiment Base in Liuhe District, Nanjing City, Jiangsu Province (32° N, 118° E) were used as experimental animals. The breeding cages used were 3-layer H-shaped structures with LED light strips at the top. Unlike laying ducks, meat ducks did not have a peak egg-laying period from 0:00 to 4:00. Their feeding, drinking, and other activities primarily occurred on well-lit days. After the lights were turned off at night, most ducks remained in the resting state.

**Figure 1:**
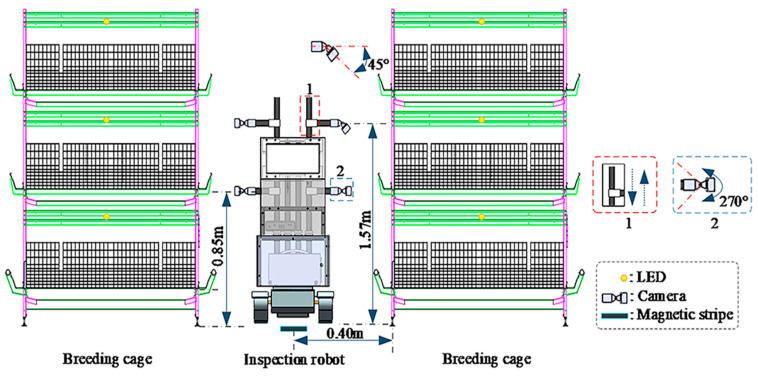
Example of the process and layout for collecting abnormal situations in cage-reared ducks.

**Figure 2:**
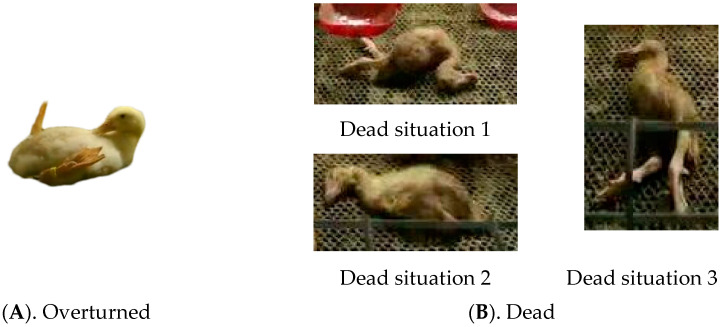
Example of abnormal cage-reared ducks.

To adequately collect samples of abnormal situations in cage-reared meat ducks, we used a duck house inspection robot equipped with a RealSense D435i depth camera (produced by Intel corporation in the United States), developed earlier, as the image-acquisition platform. The robot operated on a fixed trajectory at a constant speed from 7:00 a.m. to 8:00 p.m. on the 10th day, capturing the imagery data of cage-reared ducks at a resolution of 1280 × 720 and a frame rate of 30 fps. Subsequently, frame processing was performed manually. The camera parameters are listed in Table 1. Additionally, the layout and process of abnormal data collection for cage-reared ducks are illustrated in Figure 1, and examples of anomalies are shown in Figure 2. Moreover, to prevent the environmental stress caused by the inspection robot from affecting the ducks’ behavior, the ducks underwent adaptive training before the experiment.

Figure 2 illustrates a significant difference in the body posture of cage-reared ducks between overturned and deceased ducks. Overturned ducks typically have their backs on the ground, head and neck lifted upward near the chest, with both feet facing upward. In contrast, the deceased ducks exhibited abnormal postures, including bending of the body, leg extension, head on the ground, and closed eyes. These characteristics can be used to accurately identify and classify abnormal cage-reared ducks.

### 2.2. Preprocessing of Abnormal Cage-Reared Duck Images and Dataset Construction

**Figure 3:**
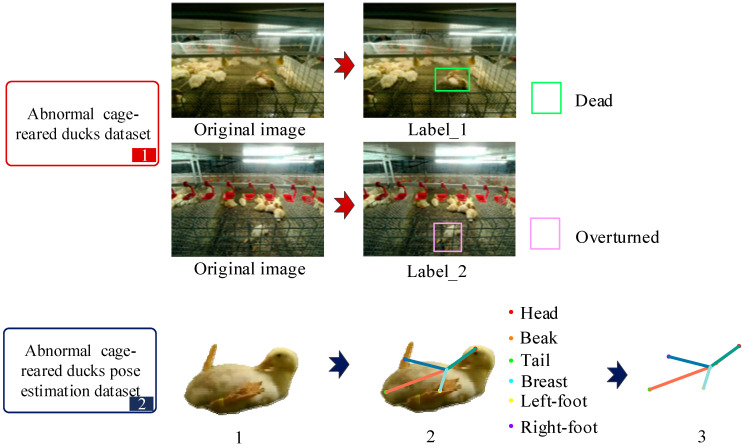
Composition of two types of dataset.

The images of abnormal cage-reared ducks were obtained through frame extraction by experienced breeders. Data augmentation techniques were employed to expand the dataset, which was ultimately normalized to 640 × 640 pixels to prevent overfitting in later stages of the model due to the limited number of image samples. Two experiments were designed: one for the identification of abnormal cage-reared ducks and another for pose estimation. Each experiment used a corresponding dataset created using SAM and Labelme image-annotation tools, following annotation rules. The definitions of abnormal situations and key parts of cage-reared ducks, as well as the composition of the abnormal cage-reared ducks dataset, are presented in Table 2 and Figure 3, respectively.

The abnormal cage-reared ducks dataset was annotated based on two typical situations: overturned and dead, with a proportion approaching 7:3 based on statistical analysis. The abnormal cage-reared ducks pose-estimation dataset defined six key points: the head, beak, chest, left foot, right foot, and tail. Following the connection rules between the head and chest, chest and left foot, chest and right foot, and chest and tail, these points characterize the posture of cage-reared ducks in abnormal situations. The distribution of the number of images in each category within the abnormal cage-reared ducks dataset is presented in Table 3.

### 2.3. Detection Method for Abnormal Situations in Cage-Reared Ducks

In 2023, Ultralytics introduced YOLOv8 as an enhancement to YOLOv5, categorizing it into five types, including n, s, l, m, and x, based on different depths and widths, with depth increasing sequentially [22]. This study selected YOLOv8n, known for its balanced detection accuracy and real-time performance, as the base network. Considering the characteristics of a large number and scale of cage-reared ducks, significant multi-scale features, and the difficulty in detecting abnormal targets due to their small size, this paper proposes a YOLOv8-ACRD (abnormal cage-reared ducks) model for identifying abnormal situations in cage-reared ducks, incorporating the GAM attention mechanism [23] and Wise-IoU loss function [24].

#### 2.3.1. YOLOv8-ACRD Network Structure

**Figure 4:**
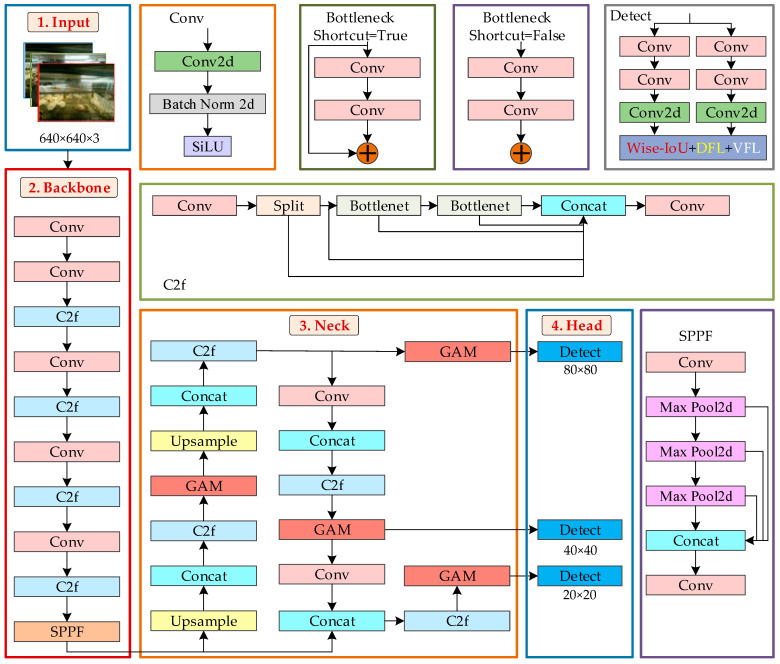
The YOLOv8-ACRD network structure.

The structure of YOLOv8-ACRD was similar to that of the original YOLOv8, consisting of four parts: the input, backbone, neck, and head. However, YOLOv8-ACRD distinguished itself by densely embedding the GAM mechanism after each C2f module in its neck. This enhancement improved the ability of the network to extract and fuse the features of abnormal cage-reared ducks, allowing the model to focus better on small-area features, such as overturned or deceased ducks in the image background, thereby enhancing recognition accuracy. Furthermore, the Wise-IoU loss function was introduced to address the data imbalance and reduce the impact of geometric factors, such as distance and aspect ratio, on the bounding box regression accuracy for abnormal cage-reared ducks. The network structure of YOLOv8-ACRD is illustrated in Figure 4.

The Input section applied mosaic data augmentation, adaptive anchor box calculation, and adaptive grayscale filling to 640 × 640 × 3 resolution cage-reared duck images before passing them to the backbone section. The Backbone performed progressive feature-extraction and generated feature maps of different scales using Convolution (Conv), Contextual Convolution (C2f), and Spatial Pyramid Pooling Fusion (SPPF) layers. The Conv layer down-sampled the images using 3 × 3 convolutional kernels with a stride of 2 and a padding of 1, followed by Batch Normalization (BN) and Sigmoid Linear Unit (SiLU) activation functions. The C2f module enhanced the gradient flow through operations such as slicing, convolution, bottleneck, and concatenation, enriching the model’s ability to learn residual features with multi-branch cross-layer connections. Finally, the SPPF layer performed predefined size transformations on the feature tensors of Conv and C2f operations. Moreover, the Backbone section output feature maps with sizes of 80 × 80, 40 × 40, and 20 × 20 to the neck section.

The Neck network in YOLOv8 incorporated the Path Aggregation Network (PANet) structure for multi-scale feature fusion, comprising certain components such as a Feature Pyramid Network (FPN) and Path Aggregation Network. The FPN constructed a feature pyramid using a top-down strategy, merging fine-grained feature maps with up-sampled coarse-grained ones to fuse feature tensors at different scales. The PANet preserved the spatial information of feature maps through bottom-up convolutional operations. Additionally, in the Neck section, multiple GAMs were densely embedded after each C2f module to allocate spatial and channel attention to the feature maps. This enhanced the network’s focus on small-scale abnormal cage-reared duck objects in the image background. After feature-fusion processing in the neck section, the feature maps of three scales (80 × 80 × 256, 40 × 40 × 512, and 20 × 20 × 512) were put out to the head network for loss calculation and detection-box filtering, thereby obtaining category and positional information of abnormal cage-reared ducks of various sizes. The YOLOv8 loss function combined three loss functions: classification loss VFL (Varifocal Loss), regression loss (CIoU), and deep feature loss (DFL). YOLOv8 ACRD introduced Wise-IoU to replace CIoU, improving the regression performance of the model in predicting bounding boxes.

#### 2.3.2. Global Attention Mechanism

**Figure 5:**
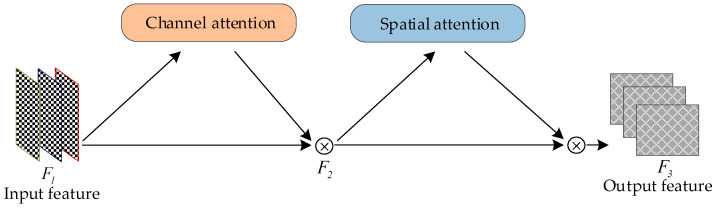
The global attention mechanism structure. Note: 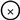 represents the multiplication of elements.

Abnormal cage-reared duck images present challenges for accurate recognition and detection because of their small object size and large background ratio. The YOLOv8-ACRD network proposed in this study aimed to enhance the focus on abnormal cage-reared duck areas in the images. This was achieved by introducing a GAM attention mechanism, which eliminated redundant information and improved the accuracy of abnormal cage-reared duck recognition under unstructured and complex environmental conditions. The GAM combined a channel attention mechanism and a spatial attention mechanism. It amplified the interaction of global dimensional features while reducing diffuse information. The structure of the GAM is illustrated in Figure 5.

**Figure 6:**
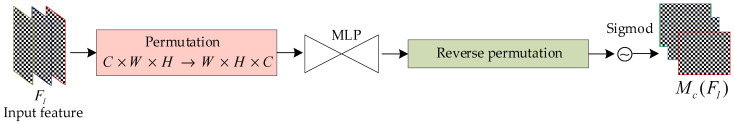
The channel attention $M_{c}$ structure. Note: 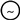 represents the Sigmoid operation.

**Figure 7:**
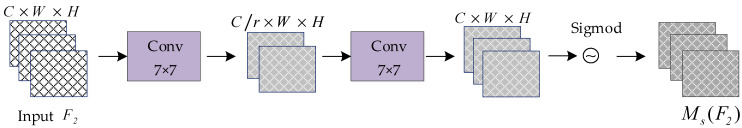
The spatial attention $M_{s}$ structure.

In Figure 5, the GAM applies channel attention correction to the input feature $F_{1}$ using two independent attention submodules, obtaining the feature $F_{2}$. Subsequently, the feature $F_{2}$ underwent the spatial attention correction to produce the output feature $F_{3}$. The channel attention and spatial attention submodules are shown in Figure 6 and Figure 7, respectively.

Figure 6 and Figure 7 illustrate the channel attention submodule, which maintained the three-dimensional information through a three-dimensional arrangement. It then enhanced the cross-dimensional channel spatial dependencies using a multilayer perceptron (MLP). The spatial sub-attention module utilized two 7 × 7 convolutional layers for spatial information fusion. Equations (1) and (2) indicate the calculations for both the channel attention and spatial attention processes.

(1) $$F_{2}=M_{c}(F_{1})⊗F_{1}$$

(2) $$F_{3}=M_{s}(F_{2})⊗F_{2}$$

In the equations, $M_{c}$ and $M_{s}$, respectively, represent the channel attention map and the spatial attention map.

#### 2.3.3. The Wise-IoU Loss Function

The abnormal cage-reared duck dataset used in this study demonstrated characteristics such as sample imbalance and long-tail distribution. Despite the small proportion of dead cage-reared ducks, death is a crucial aspect of abnormal situations in cage-reared models. The impact of the network on feature learning and inference directly affected the recognition accuracy of the model. The original YOLOv8 strategy designed a loss function based on the calculation of the intersection and union ratio between the predicted and real boxes. However, this approach ignored the recognition impact caused by differences in the number of samples in different categories. Additionally, low-quality samples in the dataset could lead to geometric factors such as target distance and aspect ratio, thereby increasing the network’s punishment for these samples and reducing the accuracy and generalization of the model. To address these issues, the Wise-IoU was introduced to replace the CIoU loss function. The Wise-IoU utilized a dynamic non-monotonic focusing mechanism to balance the data samples and reduce the excessive punishment of geometric parameters in the model. This optimization improved the training process of the abnormal cage-reared duck recognition model. The calculation process for the Wise-IoU loss value $L_{WIoU}$ is illustrated in Equations (3)–(6) below:

(3) $$L_{WIoU}=rR_{WIoU}L_{IoU}^{*}$$

(4) $$R_{WIoU}=\exp(\frac{{\left(x-x_{gt}\right)}^{2}+{\left(y-y_{gt}\right)}^{2}}{\left(w_{g}^{2}+h_{g}^{2}\right)})$$

(5) $$r=\frac{\beta }{\delta \alpha^{\beta -\delta }}$$

(6) $$\beta =\frac{L_{IoU}^{*}}{{\bar{L}}_{IoU}}$$

Here, α and δ are hyperparameters, $R_{WIoU}$ represents the geometric disparity between the predicted box and the annotation, ${\bar{L}}_{IoU}$ denotes the average dynamic Intersection over Union (IoU) loss for the predicted box, $L_{IoU}^{*}$ stands for the current IoU loss value for the predicted box, and the superscript * indicates exclusion from backpropagation. β represents the outlier value for the current predicted box, where a smaller value indicates higher anchor box quality, requiring a smaller gradient gain to be allocated. Simultaneously, smaller gradient gains can also be assigned to predicted boxes with larger outlier values, facilitating the reduction of harmful gradients from low-quality samples during network training. $w_{g}$ and $h_{g}$, respectively, represent the width and height of the box, while r represents the gain allocated to the box.

### 2.4. Method for Brightness Adjustment in Cage-Reared Duck Images

Some studies have shown that changes in light intensity directly affect duck development [25,26]. Therefore, it is necessary to adjust the brightness of LED strips according to their physiological habits to improve health and welfare. To test and analyze the generalization ability of the proposed methods for identifying abnormal situations and estimating the posture of cage-reared ducks, this study considered image brightness as a single factor. Two brightness levels, 1.25 and 0.85 times the predetermined brightness, were set to obtain images of cage-reared ducks under ‘bright’ and ‘dark’ conditions, constructing a generalization ability test dataset. Brightness adjustment of the cage-reared duck images was based on OpenCV, which converts the RGB color space to HSV. Subsequently, the V channel value, representing image brightness, was assigned a multiplier to transition between ‘bright’ and ‘dark’. Finally, the image was converted back to RGB. The process of adjusting image brightness is shown in Equation (7).

(7) $$\left\{\begin{array}{c} \mathrm{bright}=V\times 1.25\\ \mathrm{dark}=V\times 0.85 \end{array}\right.$$

### 2.5. Estimation Method for Abnormal Cage-Reared Ducks Posture

**Figure 8:**
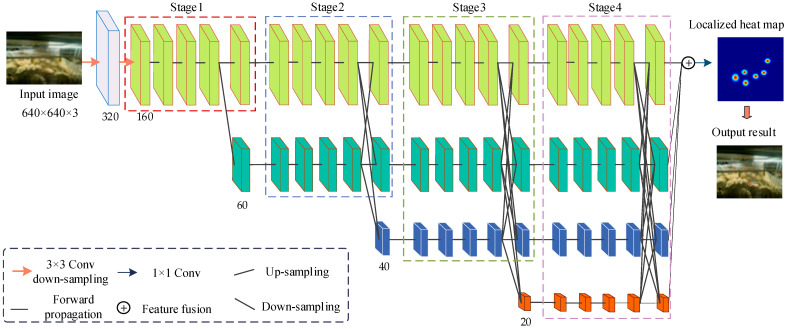
The network structure for abnormal cage-reared duck posture estimation.

Animal behavior consists of co-ordinated movements among various parts of the body. Typically, in a single behavioral state, animals may exhibit multiple postures [27]. Conducting research on posture estimation in cage-reared ducks to identify abnormal situations would be beneficial for refining their physiological status. Currently, the field of pose estimation for both humans [28,29,30] and animals [31,32,33] often employs two strategies: regression and heatmaps. Among these, the heatmap method can fully utilize spatial information from adjacent key points and joint locations, achieving high accuracy. Based on this, we used HRNet-48 [34], which is based on a heatmap, as the foundational network, and proposed an abnormal pose-estimation model for cage-reared ducks by leveraging the significant difference in the distribution of body parts between overturned and dead ducks. HRNet-48 consists of four stages with varying resolutions, parallel connections, and multi-scale feature cascades for feature analysis and localization, as illustrated in Figure 8.

### 2.6. Evaluation Criteria

To quantitatively analyze the performance of the proposed method for identifying abnormal situations in cage-reared ducks and the abnormal cage-reared ducks posture estimation model, four commonly used quantitative indicators in the field of object detection, mAP, Recall, F1 score, and OKS [35,36] were selected as the evaluation criteria, represented by Equations (8)–(11).

(8) $$\mathrm{mAP}=\frac{1}{N}\sum_{i=1}^{N}{\mathrm{AP}}_{i}$$

In the equation, N represents the total number of target categories, and ${\mathrm{AP}}_{i}$ is the average precision of the i-th category. The range of values for mAP is [0, 1], where a higher value indicates a better detection performance of the model. Additionally, in this paper, an IoU (Intersection over Union) threshold of 0.5 is set.

(9) $$\mathrm{Recall}=\frac{TP}{TP+FN}$$

In the equation, *TP* represents the number of samples correctly predicted as positive, and *FN* represents the number of samples incorrectly predicted as negative. The Recall value ranges from 0 to 1, with a higher Recall indicating a stronger ability of the model to identify positive samples.

(10) $$F1=\frac{2\mathrm{Precision}+\mathrm{Recall}}{\mathrm{Precision}+\mathrm{Recall}}$$

Here, Precision represents the proportion of true positive predictions among all positive predictions made by the model. A high F1 Score, close to 1, indicates a good balance between Precision and Recall, indicating that the model effectively identifies positives while minimizing false positives.

(11) $$\mathrm{OKS}=\frac{\sum_{i}\exp(-\frac{d_{i}^{2}}{2s^{2}k_{i}^{2}})\delta (v_{i}>0)}{\sum_{i}\delta (v_{i}>0)}$$

In the formula, i is the key point number, $d_{i}$ is the Euclidean distance between the true value and predicted value of the key point with number i, $v_{i}$ is the visibility marker of the key point with number i, invisible is 0, occluded is 1, visible is 2, δ is the Kronecker delta function, $k_{i}$ is the constant of the key point with number i, s represents a scaling factor typically defined as a percentage of the diagonal length of the bounding box.

### 2.7. Experimental Environment

The proposed methods in this paper were implemented for model training and performance evaluation on a Dell T3060 tower workstation (made in China by Dell Inc) running Windows 10 Professional. The system was equipped with an Intel i9-12900 CPU featuring a base frequency of 3.20 GHz, 128 GB of RAM, and an NVIDIA RTX 4090 GPU with 24 GB of dedicated memory. The experiments were conducted using the Python programming language within a virtual environment established based on the PyTorch deep learning framework.

### 2.8. Experimental Steps

The experiment for identifying abnormal situations and estimating posture in cage-reared ducks consisted of six steps:

1. Images of 10-day-old cage-reared ducks in the abnormal states of ‘overturned’ and ‘dead’ were collected and annotated to establish datasets for abnormal cage-reared ducks and abnormal cage-reared duck pose-estimations.
2. Based on the characteristics and experimental environment of cage-reared ducks, multiple GAM modules were densely embedded into the neck of the original YOLOv8 network, and the Wise-IoU loss function was introduced to optimize the detection performance. This led to the development of YOLOv8-ACRD, a network for recognizing abnormal situations in cage-reared ducks.
3. The proposed method, based on YOLOv8-ACRD, was tested for accuracy compared with the original YOLOv8 and evaluated against other mainstream methods to assess its effectiveness.
4. Brightness was used as a factor, with two levels (‘bright’ and ‘dark’) set to test the generalization ability of the proposed method for identifying abnormal situations in cage-reared ducks.
5. An abnormal posture estimation model based on HRNet-48 was developed by refining the identification of six key body parts in cage-reared ducks. This model was compared with other commonly used pose-estimation algorithms, and its real-time performance was evaluated.
6. The experimental results were discussed, and conclusions were drawn.

**Figure 9:**
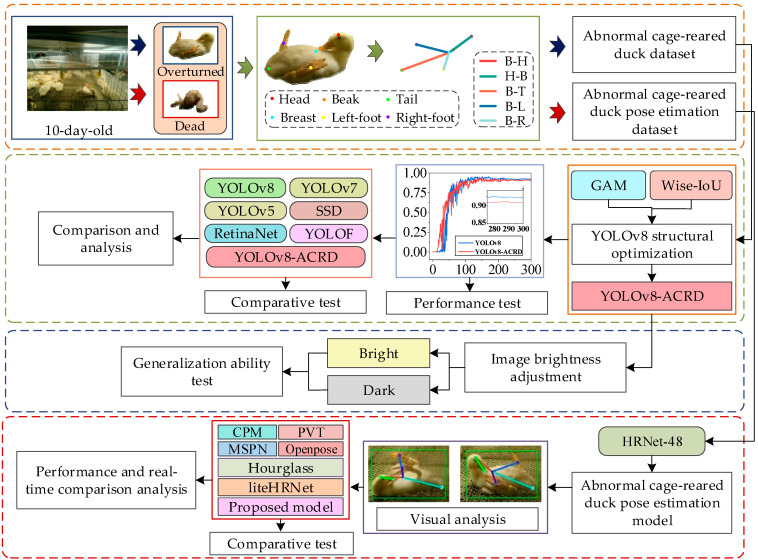
Experimental procedure for perceiving abnormal situations in cage-reared ducks.

The experimental procedure is illustrated in Figure 9.

## 3. Results

### 3.1. Abnormal Situation Recognition in Cage-Reared Ducks Based on YOLOv8-ACRD

#### 3.1.1. Acquisition of Abnormal Situation Recognition Model for Cage-Reared Ducks and Comparison of Feature Maps

**Figure 10:**
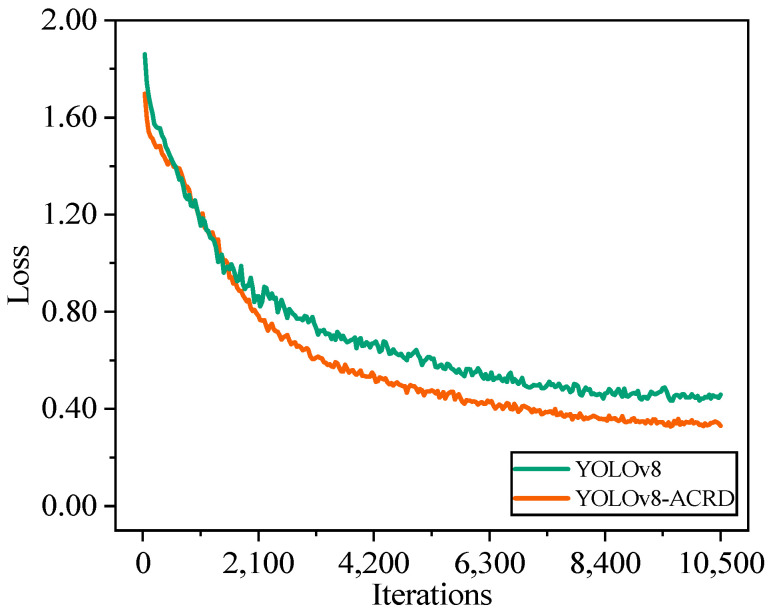
The trend of loss values with iterations during the training process of YOLOv8 and YOLOv8-ACRD.

**Figure 11:**
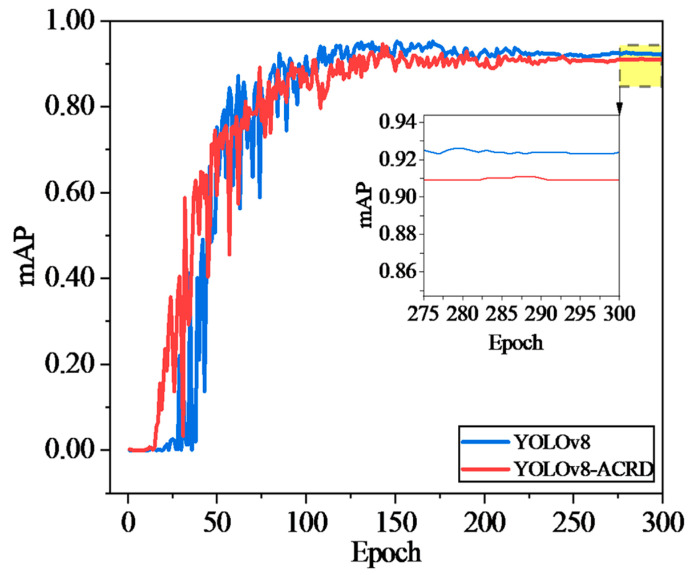
The trend of mAP values with iterations during the training process of YOLOv8 and YOLOv8-ACRD.

The training process for the optimal abnormal situation recognition model for cage-reared ducks was conducted separately on the abnormal cage-reared duck dataset based on the original YOLOv8 network and improved YOLOv8-ACRD network. To analyze and evaluate the effects of introducing GAM and Wise-IoU on model-detection accuracy, the mAP values were compared. The pretrained weights for the COCO dataset were loaded to prevent overfitting, and convergence difficulties during the COCO dataset were loaded. A Stochastic Gradient Descent (SGD) optimizer was employed for gradient descent, with the momentum set to 0.9, and the learning rate and batch size set to 0.001 and 16, respectively. Furthermore, the strategy of saving the model once per cycle was adopted, totaling 300 epochs of iterations. The trends of YOLOv8, YOLOv8-ACRD loss, and mAP values with respect to the number of iterations and epochs during this period are illustrated in Figure 10 and Figure 11, respectively.

**Figure 12:**
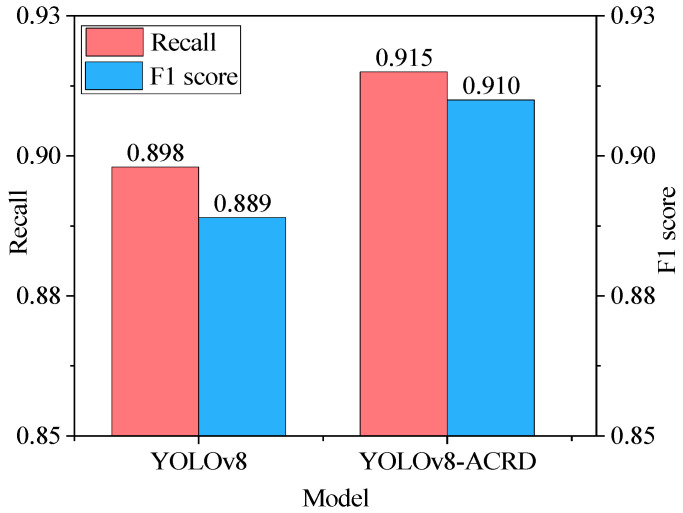
Comparison of optimal models YOLOv8 and YOLOv8-ACRD based on recall and F1 score.

In Figure 10, the loss values of YOLOv8 and YOLOv8 ACRD remained stable with the number of iterations, exhibiting a trend of rapid decrease at the beginning, gradual flattening in the middle, and convergence towards the end. However, after a rapid decrease in loss values for both models, YOLOv8-ACRD consistently exhibited lower loss than YOLOv8, a trend that continued until both models converged. In Figure 11, the trend of mAP values for YOLOv8 and YOLOv8-ACRD, with respect to epoch, mirrors that of loss, with both indicating rapid changes early on and then leveling off. Between 0 and 55 epochs, the mAP values of both models increased significantly to approximately 0.737. Subsequently, from 56 to 300 epochs, YOLOv8-ACRD consistently maintained an advantage over YOLOv8 in mAP values, consistent with the observations in Figure 10. Finally, YOLOv8 and YOLOv8-ACRD converged to the mAP values of 0.909 and 0.924, respectively. The mAP of the cage-reared duck anomaly recognition model based on YOLOv8-ACRD increased by 1.65% compared with the original YOLOv8. To further evaluate the performance differences introduced by GAM and Wise-IoU, an additional analysis was conducted based on Recall and F1 scores. The results are shown in Figure 12.

Figure 12 reveals that the Recall and F1 scores of the optimal model for detecting abnormal cage-reared ducks based on YOLOv8-ACRD increased by 0.017 and 0.021, respectively, compared with YOLOv8. This further demonstrates that YOLOv8-ACRD exhibits enhanced precision and balance in detecting instances of dead and overturned cage-reared ducks under the experimental conditions.

In summary, the results indicated that the YOLOv8-ACRD network demonstrated superior learning and inference effectiveness for detecting the distribution patterns of overturned and dead ducks compared with YOLOv8. This confirmed the positive impact of employing dense GAM embedding and introducing the Wise-IoU loss function to enhance detection accuracy. Consequently, YOLOv8-ACRD was selected as the optimal model for subsequent experiments to test anomaly recognition and generalization abilities in cage-reared ducks.

**Figure 13:**
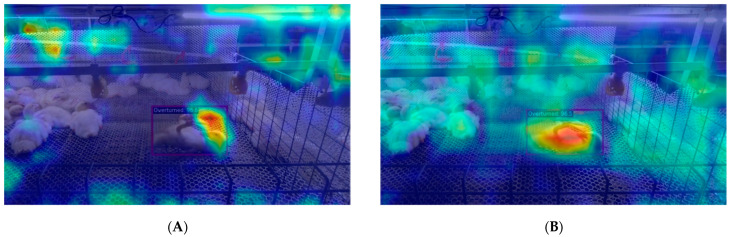
The visualization comparison of maximum activation feature maps for the neck in YOLOv8 and YOLOv8-ACRD. (**A**). YOLOv8, (**B**). YOLOv8-ACRD.

Figure 13 presents the visualization of the feature maps with the maximum activation in the three output channels of the neck section for both the YOLOv8 and YOLOv8-ACRD networks overlaid on the original images. YOLOv8-ACRD demonstrated more precise localization in identifying the features of cage-reared ducks in the overturned states, compared to YOLOv8. It can exclude interference from other similar features and accurately focus on target ducks in the images. In contrast, YOLOv8 demonstrated deviations in the attention area and actual position of features related to overturned cage-reared ducks. Although it covered the duck’s body, some features extended beyond this area. These observations further validated that the proposed YOLOv8-ACRD cage-reared duck abnormal recognition model had an accuracy advantage over the original YOLOv8.

#### 3.1.2. Recognition of Abnormal Situations in Cage-Reared Ducks

**Figure 14:**
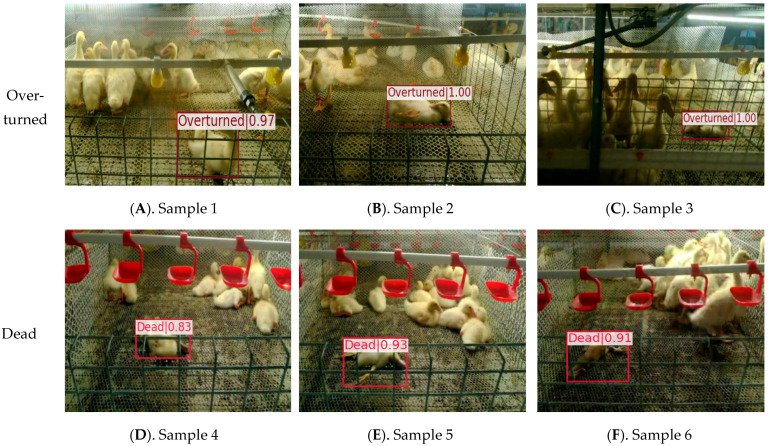
Recognition results of abnormal situations in cage-reared ducks based on YOLOv8-ACRD.

Using the proposed YOLOv8-ACRD model to identify abnormal situations in cage-reared ducks, the dataset was analyzed to identify overturned and dead situations. The selected results are shown in Figure 14.

In Figure 14, cage-reared duck samples 2–6 in the overturned or deceased states were partially occluded to varying degrees by other ducks or cage meshes. For instance, in sample 2, the head of the cage-reared ducks was partially obscured, whereas in samples 3 and 5, the bodies were covered with a cage mesh. However, the proposed model correctly identified anomalous states with high confidence levels. The bounding box positioning was accurate with no instances of false positives or false negatives. Furthermore, the different sample images exhibited varying degrees of depth-of-field, and there were significant differences in multi-scale features among the ducks. Despite this, the proposed model maintained a high level of recognition accuracy, indicating its robust performance. Finally, the recognition model based on YOLOv8-ACRD for anomalous situations in cage-reared ducks achieved Average Precision (AP) values of 0.913 and 0.935, respectively, for recognizing overturned and dead situations in the validated subset of the abnormal cage-reared duck dataset.

#### 3.1.3. Comparison and Analysis

**Figure 15:**
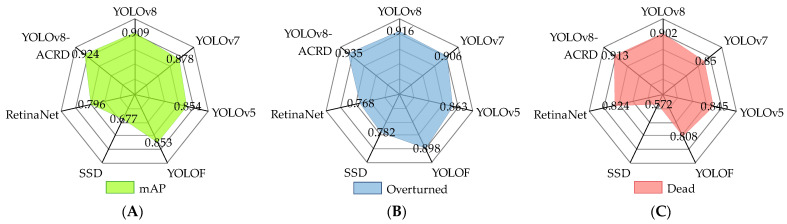
Comparative experimental results. (**A**). Distribution of mAP values for each model; (**B**). Distribution of AP values for cage-reared duck overturned situation recognition; (**C**). Distribution of AP values for cage-reared duck dead situation recognition.

Object-detection algorithms based on deep learning are continuously advancing in the field of computer visualization. However, these algorithms often vary in effectiveness when applied to different objects and environments. This study evaluated the effectiveness of the proposed anomalous situation-recognition method based on YOLOv8-ACRD, compared with other mainstream object-detection algorithms. To satisfy the requirements of detection accuracy and real-time performance for cage-reared duck inspection robots, a comparative experiment was conducted, focusing on single-shot object-detection algorithms capable of balancing recognition accuracy and speed. The experiments compared YOLOv8, YOLOv7 [37], YOLOv5 [38], YOLOF [39], SSD [40], and RetinaNet [41]. To maintain environmental consistency, the same computer platform, compilation environment, and training hyperparameters were used, which were consistent with YOLOv8-ACRD. The comparison results, including mAP values for the abnormal cage-reared duck dataset and AP values for the two abnormal situations, are illustrated in Figure 15.

The mAP values for abnormal situations in cage-reared ducks indicated that YOLOv8-ACRD improved by 0.015, 0.046, 0.070, 0.071, 0.247, and 0.128, compared with YOLOv8, YOLOv7, YOLOv5, YOLOF, SSD, and RetinaNet, respectively. The overall performance difference between YOLOv5 and YOLOv8 was not significant. However, RetinaNet and SSD exhibited relatively low performances, with SSD achieving only 0.677 mAP. This indicated that, in this experimental environment, the proposed model performed best, whereas SSD was the least effective and not suitable for identifying abnormal situations in cage-reared ducks. This disparity may be attributed to the structure of the SSD, which, despite utilizing deep convolutional neural networks to extract features of abnormal cage-reared ducks, lacked sufficient multi-scale feature fusion compared to other networks. Furthermore, the absence of attention mechanisms in SSD resulted in insufficient focus on important features, especially in cases where cage-reared duck images had complex backgrounds and significant variations in duck phenotypes, making the accurate localization of object duck features challenging. Analysis of the changes in AP values for the recognition of dead and overturned cage-reared ducks in Figure 14B,C reveals a consistent pattern: YOLOv8, YOLOv7, YOLOv5, YOLOF, SSD, and the proposed model were better at recognizing overturned situations than dead situations. However, RetinaNet demonstrated the opposite trend, with an AP value of 0.824 for dead ducks, which was 0.056 higher than that for overturned ducks. Furthermore, compared to the aforementioned six models, the proposed model enhanced the recognition AP values for overturned and dead ducks by 0.011, 0.019, 0.063, 0.029, 0.068, 0.072, 0.105, 0.037, 0.341, 0.153, 0.089, and 0.167, respectively. Overall, in the experimental environment, the proposed model demonstrated a precision advantage over other mainstream object-detection algorithms in recognizing both types of abnormal situations in cage-reared ducks.

### 3.2. Perception of Abnormal Situations in Cage-Reared Ducks under Different Lighting Conditions

**Figure 16:**
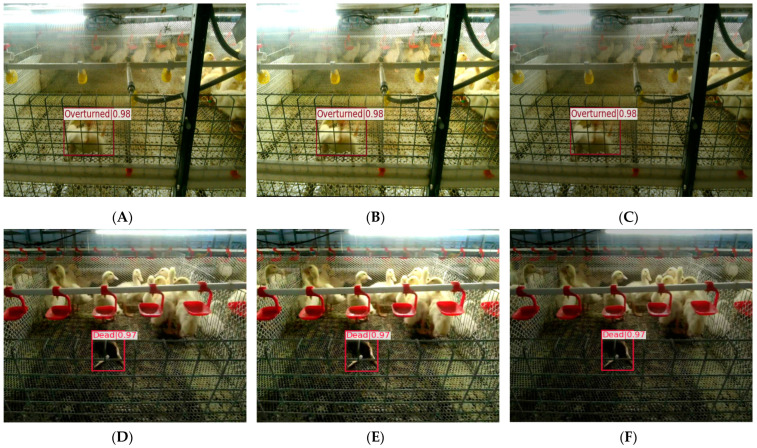
Generalization ability test results of cage-reared ducks abnormal situation recognition model based on YOLOv8-ACRD. (**A**). Sample 1; (**B**). Result after brightening sample 1; (**C**). Result after darkening sample 1; (**D**). Sample 2; (**E**). Result after brightening sample 2; (**F**). Result after darkening sample 2.

Image acquisition of cage-reared ducks was conducted under a predetermined light intensity of LED light sources with no specific requirements for direct or back lighting. Although the proposed model achieved high accuracy in recognizing abnormal situations in this scenario, differences in lighting intensity existed across cage-reared-duck breeding facilities at different rearing stages. The recognition performance of the proposed model under varying lighting conditions was unclear, necessitating the testing of its generalization ability. To assess this, 50 randomly selected original images containing overturned and dead cage-reared ducks were used. The brightness adjustments were applied to these images to create a total of 100 images categorized as ‘bright’ and ‘dark’, using the brightness adjustment method described in Section 2.4. Subsequently, the generalization ability of the model was tested based on these images. The selected recognition results are shown in Figure 16.

The results shown in Figure 15 reveal significant differences in the external appearance of cage-reared ducks under varying lighting conditions. For instance, the high brightness of head feathers caused feature loss in samples 1 and 2, and the dim lighting led to tail feathers blending with the background, causing confusion. However, the proposed abnormal situation recognition model based on YOLOv8-ACRD effectively eliminated these interferences and accurately detected the overturned and dead cage-reared ducks. These findings suggest that changes in image lighting intensity did not significantly affect the performance of the model, as presented in this paper. After adjusting the ambient brightness for recognition of abnormal situations in caged-duck breeding, the model in this study achieved an mAP value of 0.881. This indicated its applicability in recognizing abnormal cage-reared ducks under different lighting intensities, showing its strong generalization ability.

### 3.3. Abnormal Cage-Reared Duck Pose Estimation

#### 3.3.1. Results of Abnormal Cage-Reared Duck Pose Estimation

**Figure 17:**
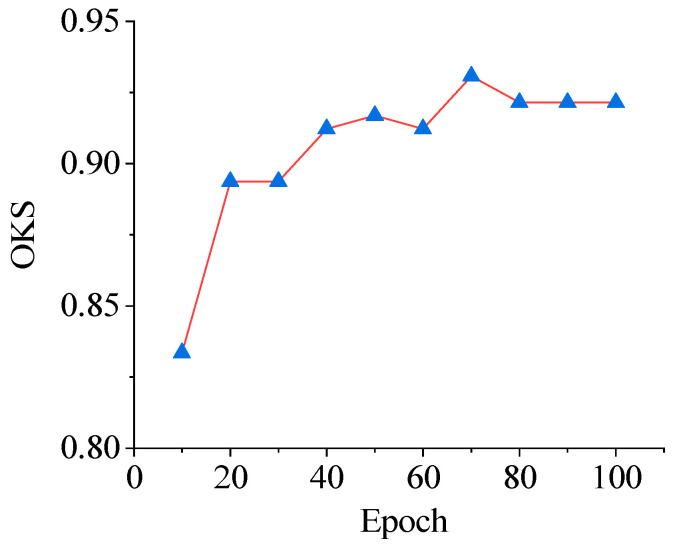
Trend of OKS values with epoch variation based on HRNet-48 abnormal cage-reared ducks pose-estimation model.

In the initial stage of the experiment, the focus was on recognizing two types of abnormal situations in cage-reared ducks: overturned and dead. The approach centered on six key points: the duck’s head, beak, chest, tail, left foot, and right foot, following the preset connection rules. An abnormal cage-reared duck pose-estimation model was developed based on an HRNet-48 network. During the model training process, the Adam optimizer was used for gradient descent, with a learning rate of 0.0001. The model was saved every 10 epochs for 100 iterations. Throughout this training period, the trend of the OKS value with respect to the epochs was monitored, as shown in Figure 17.

**Figure 18:**
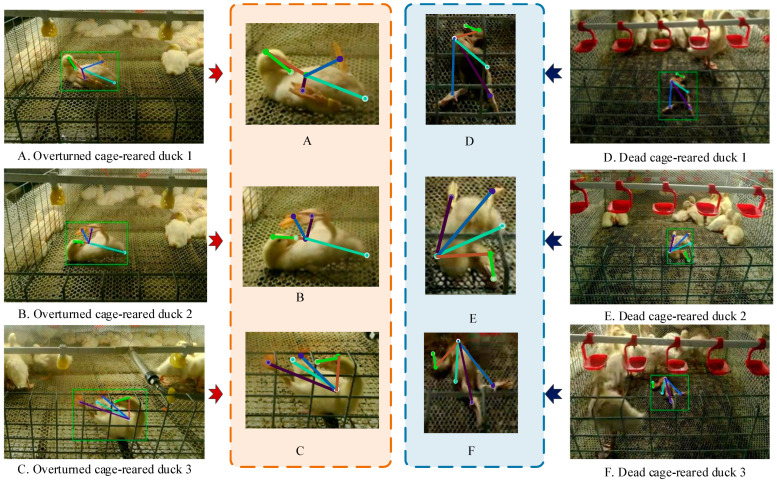
Recognition results of abnormal cage-reared ducks pose-estimation model based on HRNet-48.

Figure 16 shows that the OKS value of the abnormal cage-reared duck posture estimation model increased rapidly during the initial 0–20 epochs. Subsequently, the rate of increase decelerated, reaching its peak at epoch 70, before decreasing and stabilizing at approximately 0.921, indicating convergence. Therefore, this model was selected for subsequent visualization of abnormal cage-reared duck pose estimations. The results are shown in Figure 18.

The visualization effect was poor because of the small pixel area occupied by the target ducks in the abnormal states. Therefore, the region of interest was zoomed in proportion. In Figure 18, significant differences in the postures of cage-reared ducks in the same abnormal state were evident, despite interference from the cage mesh. Additionally, dead ducks are often trampled by other ducks, resulting in varying degrees of surface contamination that distinctly alters their color compared to overturned ducks. Meanwhile, the differences in key features among the dead ducks were minimal. None of these conditions is conducive to accurately estimating the posture of abnormal ducks. For example, in Figure 18, the limb distributions of dead cage-reared ducks 1 and 2 differed, whereas the colors of cage-reared ducks 1 and 3 closely matched the background. Furthermore, some of the features of overturned cage-reared duck 3 were obscured by the cage mesh. However, the proposed model accurately detected and classified six key points, such as the duck’s head and beak, without any missed or false detection. The key points can be assembled into a duck trunk in a predetermined association sequence without any errors. These findings indicate that the proposed abnormal cage-reared duck pose-estimation model could accurately detect overturned and dead cage-reared duck poses and key body parts, while also demonstrating a certain degree of interference resistance.

#### 3.3.2. Comparison and Analysis

**Figure 19:**
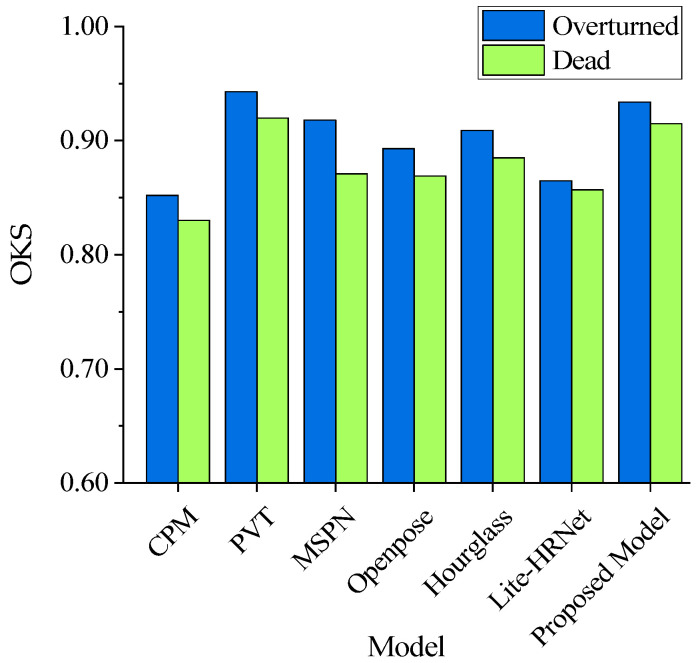
Comparison results of OKS for abnormal cage-reared duck pose estimation.

**Figure 20:**
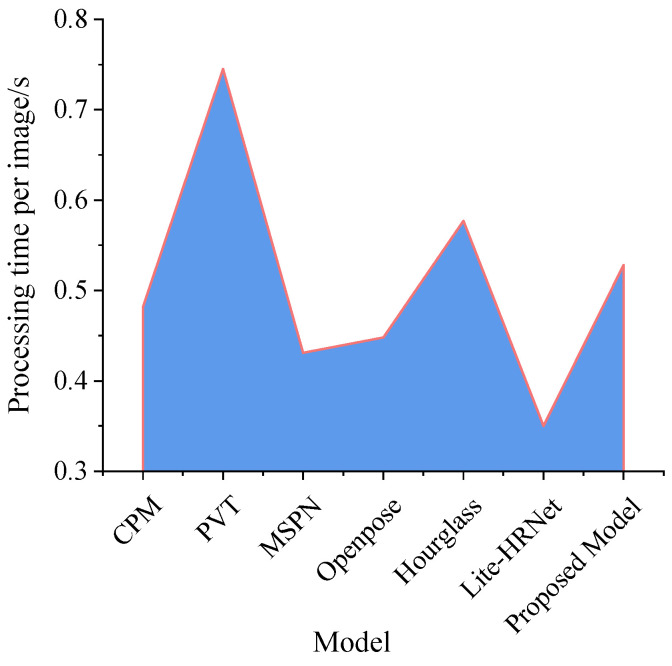
Real-time performance comparison results.

Recent research has made significant progress in refining methodologies for detecting key body parts and poses, building on advancements in animal and human behavior detection. To compare the proposed abnormal cage-reared duck pose-estimation model with other mainstream methods, this study introduced CPM [42], PVT [43], MSPN [44], Openpose [45], Hourglass [46], and liteHRNet [47]. To evaluate the impact of cage-reared duck overturned and dead postures on model accuracy, this study categorized the images containing overturned and dead cage-reared ducks from the abnormal cage-reared duck pose-estimation dataset, and conducted targeted performance testing. The comparative experiments maintained consistency in the compilation environment, hyperparameters, deep-learning frameworks, and computer models used. The optimal models of the six aforementioned methods and the model proposed in this study were compared based on the OKS, as shown in Figure 19. The inspection of the physiological status of cage-reared ducks by breeding robots requires a pose-estimation model that not only has high recognition accuracy but also exhibits excellent real-time performance. Therefore, focusing on the processing time of a single image, this study further compared the real-time performance with the aforementioned methods for the abnormal cage-reared duck pose-estimation dataset. The real-time performance comparison results are shown in Figure 20.

Based on the comparison results in Figure 19, CPM, PVT, MSPN, Openpose, Hourglass, liteHRNet, and the model proposed in this study consistently demonstrated a higher accuracy in detecting the overturned poses of cage-reared ducks than in detecting the dead poses. This difference may be attributed to the fact that, when a duck was overturned, its key body parts were more fully presented in the image, and there was less mutual occlusion compared with the dead situation. This facilitated the model’s extraction and learning of multi-dimensional features of abnormal cage-reared ducks, thereby aiding key point classification and position inference. Additionally, the OKS values of the above models for the overturned and dead postures were 0.852, 0.830, 0.943, 0.920, 0.918, 0.871, 0.893, 0.869, 0.909, 0.885, 0.865, 0.857, 0.934, and 0.915, respectively. PVT achieved the highest accuracy, followed closely by the model in this study, with negligible differences of only 0.009 and 0.005, respectively. This minor difference could be attributed to the PVT’s application of a self-attention mechanism, which enhanced the model’s ability to focus on key features based on the transformer architecture. This mechanism enables more accurate feature localization compared to HRNet-48, which relies on a multi-resolution channel-cascading strategy. In contrast, CPM exhibited the lowest accuracy. This suggests that relying solely on convolutional forms for inferring key point categories and positions of abnormal cage-reared ducks was not suitable for the experimental environment of this study.

In the real-time performance comparison results shown in Figure 20, the single image processing times of the models were 0.482, 0.745, 0.431, 0.448, 0.577, 0.350, and 0.528 s, respectively. Among these, Lite HRNet demonstrated the best real-time performance, being 0.178 s faster than the model in this study, albeit with slight decreases in accuracy of 0.069 and 0.058, respectively. This indicates that, although the lightweight HRNet network could reduce the model inference time, it may compromise the accuracy improvement. Moreover, compared with PVT, the model proposed in this study reduced the processing time by 0.217 s. Given the minimal difference in accuracy between the two models, this suggested that, for estimating abnormal cage-reared duck poses, the model proposed in this study achieved a balance between high accuracy and excellent real-time performance, indicating the best comprehensive detection ability.

## 4. Discussion

During the exploration of the stereoscopic cage-reared mode for meat ducks, timely detection and identification of abnormal ducks are crucial for reducing the risk of disease transmission, ensuring group health and breeding welfare, and promoting the stable development of the poultry industry. Utilizing artificial intelligence technology for accurate and real-time perception of abnormal cage-reared ducks is a key trend in the industry’s stable development. However, current research on poultry behavior detection has primarily focused on meat/egg chickens, and there is a lack of technology and theoretical research on detecting abnormal behavior in meat ducks. This study addressed this gap by focusing on the abnormal states of overturning and death that often occur in 10-day-old cage-reared ducks. Using computer vision technology, a method for identifying abnormal situations in cage-reared ducks was proposed, and further research was conducted on the estimation method of abnormal cage-reared duck posture, achieving accurate and real-time detection of two typical abnormal states. The following three points of discussion are presented based on the experimental process and results.

### 4.1. Influence of Different Abnormal States of Cage-Reared Ducks on Model Recognition and Pose-Estimation Accuracy

In this experiment, we proposed a method for the recognition of abnormal cage-reared ducks, focusing on the two typical abnormal states of overturned and dead, based on YOLOv8-ACRD. We further refined this approach by exploring an abnormal cage-reared duck posture estimation model based on HRNet-48, focusing on six key points, including the head, beak, chest, tail, left foot, and right foot. Our research demonstrated the effectiveness and accuracy of these models. We also discovered that both proposed models exhibited a pattern of higher detection accuracy for overturned cage-reared ducks than for dead ducks. This pattern was also observed in performance comparison experiments with other mainstream methods. This phenomenon may be attributed to the fact that, when a cage-reared duck is overturned, its head, legs, and other body parts are not obstructed. In contrast, in the case of death, the duck’s body is covered to varying degrees by the trampling and attacks of other ducks, making feature extraction and localization more challenging, and resulting in differences in detection accuracy. This finding could serve as a reference for research focusing on the recognition of physiological states of livestock and poultry based on specific body shapes and postures.

### 4.2. Impact of Introducing Attention Mechanism and Optimized Loss Function on Model Performance

Numerous studies have shown that tailoring attention mechanisms, changing loss functions, or deepening network architectures based on different experimental subjects and environments can enhance model performance. In this study, we built upon YOLOv8 as the base network and embedded the GAM after each c2f module in its neck section to enhance the focus on regions of abnormal cage-reared ducks in the images. Additionally, we replaced the CIoU loss function with Wise-IoU to optimize the model-training process, resulting in the proposed YOLOv8-ACRD for detecting abnormal situations in cage-reared ducks. The experimental results demonstrated that the abnormal situation-recognition model for cage-reared ducks based on YOLOv8-ACRD significantly improved detection accuracy compared to the original YOLOv8. This finding suggests that, in specific object-detection tasks, selective attention mechanisms or other optimization methods based on the properties of the experimental object can improve model performance and enhance detection effectiveness.

### 4.3. Limitations and Future Directions

Extensive experiments have demonstrated that the proposed method and pose-estimation model for identifying and estimating the poses of cage-reared ducks in overturned and dead situations achieve accurate detection. The proposed approach exhibited excellent generalization ability and robustness, accurately detecting the key parts of the abnormal cage-reared duck body and forming correct pose expressions. It also demonstrated superior overall performance compared to other commonly used methods. However, this study had certain limitations.

(1) Ducks are inherently sensitive and susceptible to stress, making them prone to a range of bacterial and viral diseases. Although various types of abnormal situations can occur, this study specifically focused on detecting two common scenarios: overturned and dead ducks. A gap exists between the diverse range of abnormal conditions observed in real-world cage-reared ducks and those addressed in this study. Future research could incorporate thermal infrared sensing, audio processing, and hyperspectral/near-infrared technologies. A more comprehensive method for identifying various abnormalities in cage-reared ducks can be developed by integrating temperature, sound, and fecal spectral information through multisource data fusion.
(2) The abnormal cage-reared duck dataset and abnormal cage-reared duck pose-estimation dataset were labeled manually, a labor-intensive process. Future research should concentrate on semi-supervised approaches to enhance the model’s performance, with reduced manual effort and data requirements.
(3) As ducks aged, their appearance and shape changed significantly. The experimental subjects in this study were limited to 10-day-old ducks. Future research should incorporate age gradients to improve the robustness of this model.
(4) This study did not address the simultaneous detection and classification of multiple abnormal ducks, nor did it effectively estimate the posture of heavily obscured ducks. Given the high-density nature of poultry farming, future research will focus on the multi-target detection of abnormal ducks in such scenarios, as well as on feature generation and completion.

## 5. Conclusions

This study introduced a method for recognizing abnormal situations in cage-reared ducks based on YOLOv8-ACRD. The method achieved accurate detection of overturned and dead situations, with an mAP of 0.924, surpassing the original YOLOv8 by 1.65%. It also effectively handled the recognition interference caused by changes in lighting conditions, demonstrating excellent generalization and robustness. Compared to other methods, this approach demonstrated superior overall performance. Additionally, by refining the structure of the abnormal cage-reared duck body and focusing on six key parts, an abnormal caged-duck pose-estimation model based on HRNet-48 was proposed. The model achieved an OKS value of 0.921, accurately detecting the key points of the cage-reared ducks and generating correct posture expressions. Furthermore, it demonstrated excellent real-time performance, surpassing other mainstream pose-estimation models in terms of accuracy and efficiency. Moreover, both of the proposed models consistently demonstrated lower perception accuracy for dead cage-reared ducks than for overturned ducks. This method could offer technical support for enhancing the duck cage-reared mode, ensuring animal welfare, and serving as a reference for the intelligent breeding of other poultry animals.

## Figures and Tables

**Table 1:** Partial parameters of RealSense D435i.

Attribute	Parameter
Resolution and FPS	1280 × 720 30 fps
Dimensions (mm)	90 × 25 × 25
RGB FOV (H × V)	69° × 42°
Depth FOV	87° × 58°
Ideal range (m)	<3
Interface type	USB 3

**Table 2:** Definition of abnormal situations and key body parts of cage-reared ducks.

Dataset	Labels	Abnormal Situations and Key Points Definitions
Abnormal cage-reared ducks dataset	Overturned	Cage-reared ducks exhibit a supine posture with both feet pointing upwards, their backs pressed against the ground, heads and necks inclined away from the ground, with a tendency to sway from side to side.
	Dead	Cage-reared ducks adhere to the ground in a deformed posture, remain stationary, with stains covering their feathers.
Abnormal cage-reared ducks pose-estimation dataset	Head	Cage-reared ducks crown feather region
	Beak	Cage-reared ducks upper beak region
	Breast	Cage-reared ducks breast feather region
	Tail	Cage-reared ducks tail feather region
	Left foot	Facing the cage-reared ducks, the left palm area of the cage-reared ducks
	Right foot	Facing the cage-reared ducks, the right palm area of the cage-reared ducks

**Table 3:** The distribution of the abnormal cage-reared ducks dataset.

Abnormal Cage-Reared Ducks Dataset		
	Train	Test
Overturned	3154	603
Dead	1297	237

## Data Availability

Data are contained within the article.
